# Imaging Properties and Tumor Targeting of ^68^Ga-NeoBOMB1, a Gastrin-Releasing Peptide Receptor Antagonist, in GIST Patients

**DOI:** 10.3390/biomedicines10112899

**Published:** 2022-11-11

**Authors:** Leonhard Gruber, Clemens Decristoforo, Christian Uprimny, Peter Hohenberger, Stefan O. Schoenberg, Francesca Orlandi, Maurizio Franco Mariani, Claudia Manzl, Maria Theresia Kasseroler, Herbert Tilg, Bettina Zelger, Werner R. Jaschke, Irene J. Virgolini

**Affiliations:** 1 Department of Radiology, Medical University Innsbruck, Anichstrasse 35, 6020 Innsbruck, Austria; 2 Department of Nuclear Medicine, Medical University Innsbruck, Anichstrasse 35, 6020 Innsbruck, Austria; 3 Division of Surgical Oncology and Thoracic Surgery, Medical Faculty Mannheim, Heidelberg University, Ludolf-Krehl-Straße 13-17, 68167 Mannheim, Germany; 4 Department of Radiology and Nuclear Medicine, University Medical Centre Mannheim, Heidelberg University, Ludolf-Krehl-Straße 13-17, 68167 Mannheim, Germany; 5 Advanced Accelerator Applications, Via Ribes, 10010 Colleretto Giacosa, Italy; 6 Division of Pathology, Medical University Innsbruck, Anichstrasse 35, 6020 Innsbruck, Austria; 7 Department of Internal Medicine V, Medical University Innsbruck, Anichstrasse 35, 6020 Innsbruck, Austria; 8 Department of Internal Medicine I, Medical University Innsbruck, Anichstrasse 35, 6020 Innsbruck, Austria

**Keywords:** GIST, PET/CT, ^68^Ga-NeoBOMB1, GRPR, phase IIa study

## Abstract

Background: Gastrin-releasing peptide receptors (GRPRs) are molecular imaging targets in multiple malignancies. Recently, NeoBOMB1, a ^68^Ga-labelled antagonist to GRPRs, was developed for PET. Here we report the outcome of a Phase I/IIa clinical trial (EudraCT 2016-002053-38) describing diagnostic properties and covariates influencing uptake of ^68^Ga-NeoBOMB1 in oligometastatic gastrointestinal stromal tumor (GIST) patients. Methods: Nine patients with advanced GIST using PET/CT (computed tomography) were included. After kit-based ^68^Ga-NeoBOMB1 preparation with a licensed ^68^Ge/^68^Ga generator, 3 MBq/kg body weight were injected intravenously. PET/CT included dynamic and static PET scans 5, 12 and 18 min and 1, 2, and 3–4 h post injection (first six patients) and static PET scans 2 and 3–4 h post injection (last three participants). Tumor targeting was assessed on a per-lesion and per-patient basis. Results: Six patients showed visible radiotracer uptake in at least one tumor lesion. Seventeen out of 37 tumor lesions exhibited significant ^68^Ga-NeoBOMB1 uptake (median SUV_max_ 11.8 [range 2.8–51.1] 2 h p.i. and 13.2 [range 2.5–53.8] 3–4 h p.i) and improved lesion-to-background contrast over time. Five lesions (13.5%) were identified only by ^68^Ga-NeoBOMB1-PET, with no correlation on contrast-enhanced CT. Three patients showed no radiotracer accumulation in any lesions. Tracer uptake correlated with male sex (*p* < 0.0001), higher body mass index (*p* = 0.007), and non-necrotic lesion appearance (*p* = 0.018). There was no association with whole-lesion contrast enhancement, hepatic localization, mutational status, or disease duration. Conclusions: ^68^Ga-NeoBOMB1-PET exhibits variable tumor uptake in advanced-stage GIST patients, correlating with lesion vitality based on CT contrast uptake, opening the possibility of a theragnostic approach in selected cases.

## 1. Introduction

Gastrointestinal stromal tumors (GIST) are rare sarcoma subtypes [1,2,3] likely arising from the interstitial cells of Cajal [4] after mutations to the c-kit gene affecting the tyrosine kinase receptor [5] or the platelet-derived growth factor receptor alpha (PDGF-Rα) [6]. Incidence rates range from 4.3 to 22 per million, depending on study and population examined [7]. Median age at diagnosis is 65 years (reported range 10–100 years) with primarily gastric tumor localization. Small intestinal, colonic, or rectal localizations are rare [7]. A total of 81.3% of patients are symptomatic at first diagnosis with abdominal pain, gastrointestinal bleeding and obstruction or other non-specific complaints [7]. A total of 80–85% of GISTs present localized disease at first diagnosis, but metastasis is a frequent phenomenon over the course of the disease in up to 85% of patients [8].

As GISTs rarely respond to treatment with standard cytotoxic chemotherapy [1,4], surgery had been the therapeutic mainstay before the advent of tyrosine-kinase inhibitors (TKI). TKI such as imatinib, sunitinib (second-line), and regorafenib (third-line) have significantly increased overall survival [9], but up to 80% of patients still develop TKI resistance over time [10], which is often based on mutations changing the conformity of the receptor, leaving little effective therapeutic options for these patients. To date, second-line chemotherapy offers a median progression-free survival of 6–9 months, while external beam radiotherapy is limited by organs at risk in the proximity of the tumor [4]. Alternative approaches such as endoradiotherapy, aggressive metastasectomy [11] or minimally invasive ablation techniques [12] are effective for local control, yet do not provide a systemic treatment approach and may not reliably improve overall survival [4].

While initial diagnosis and follow-up strongly rely on computed tomography (CT) and magnetic resonance imaging (MRI), imaging can be unspecific and of moderate sensitivity to small tumor lesions, especially of the stomach as well as peritoneal lesions smaller than 5 mm [13]. Follow-up is complicated by the fact that RECIST criteria (Response evaluation criteria in solid tumors) are inappropriate because tumor size is no definitive hallmark of therapy response [13].

While positron-emission tomography based on ^18^FDG-PET allows for better sensitivity in the initial staging process and assessment of tumor response after treatment [13], it is highly unspecific. GIST cells have been described to exhibit high expression levels of surface peptide receptors, in particular Gastrin Releasing Peptide receptors (GRPRs) [14], regardless of disease stage or treatment [15], suggesting GRPR targeting as a viable option for diagnostic imaging. Several clinical studies have already shown expression of GRPRs in a variety of tumor entities such as prostate [16,17,18,19,20], breast cancer [21], glioma [22,23], and GIST [14]. ^68^Ga-NeoBOMB1, now called ^68^Ga-NeoB—a novel DOTA-coupled GRPR antagonist derived from native bombesin by C-terminal Leu^13^-Met^14^-NH_2_ modification—was identified to be a suitable candidate for GIST targeting [24] within the EU-FP7 project Closed-loop Molecular Environment for Minimally Invasive Treatment of Patients with Metastatic Gastrointestinal Stromal Tumours (‘MITIGATE’, grant agreement number 602306). ^68^Ga-NeoBOMB1 has been shown to possess high affinity for the GRPR expressed on the cell surface of a human prostate cancer model (PC-3) (6–14).

Here we report the outcome of a prospective clinical Phase I/IIa pilot study on tumor targeting and imaging data in patients with advanced GIST under current or previous TKI treatment included in this pilot study (n = 9), complementing our initial report on the safety, tolerability, and pharmacokinetics of ^68^Ga-NeoBOMB1 from the first six patients [25].

## 2. Materials and Methods

### 2.1. Study Approval and Registration

Study approval was granted on 25 July 2016 by the local Ethical Review Board (Ethics Committee Medical University Innsbruck) and by the Austrian Competent Authority (Bundesamt für Sicherheit im Gesundheitswesen) on 28 November 2016. Monitoring duties were performed by the local Clinical Trial Centre (KKS, MUI). Study initiation was performed in December 2016 after registering within EudraCT (2016-002053-38) and ClinicalTrials.gov (accessed on 19 July 2019) (NCT02931929). All participants provided written informed consent.

### 2.2. Study Goals

The primary endpoints were qualitative and quantitative evaluation of ^68^Ga-Ne BOMB1 uptake in known GIST lesions; secondary endpoints were the identification of covariates influencing the uptake in light of a potential diagnostic and potentially therapeutic application.

### 2.3. Subjects, Study Plan and Safety Assessment

Overall, nine patients with advanced GIST—i.e., suffering from surgically incurable metastasized disease—were enlisted in both phases of the study, with phase I (six participants) following a broader approach with safety, tolerability, and pharmacokinetic analysis in mind [25] and phase II (an additional three participants) focusing on tumor targeting and imaging timeframes. According to the study plan [25], at least 50% of patients were required to exhibit a first-, second-, or third-line TKI resistance—defined by disease progress under treatment. Clinical and imaging data were used to classify disease progression (usually based on Choi criteria [26]). To minimize a preselection bias of participants with greater disease burden, four patients with a stable disease were enrolled. Furthermore, patients had to be older than 21 years and have a Karnofsky index > 70%. Patients with a known pregnancy, severely impaired renal function (eGFR < 45 mL/min/1.72 m^2^), known haematotoxicity grade 2 or higher, other known malignancies diagnosed within the last five years (except for melanoma and uterine cervical malignancy), intolerance to one of the used tracer compounds or bladder outflow obstructions, or severe incontinence were excluded from the study. Contraception during the study period was mandatory for male and female participants. A detailed table on the inclusion and exclusion criteria is available from [25]. For further details on disease status and treatment at the time of study inclusion, please refer to Table 1.

The Common Terminology Criteria for Adverse Events v5.0 (NIH; Bethesda, MD, USA) were used to document and grade severity and causal correlation to the application of ^68^Ga-NeoBOMB1 for adverse (AE) and severe adverse events (SAE).

Participants were screened and enrolled at least 24 h prior to the tracer administration (visit 0), where medical history, physical status, and inclusion and exclusion criteria were reviewed. A urine dipstick test was performed in premenopausal female participants to exclude a potential pregnancy.

Prior to the tracer application (visit 1), a review of the inclusion and exclusion criteria as well as medical history and physical status was performed. For a thorough description of the study plan, safety measures and evaluation of safety, tolerability, and pharmacokinetics, please refer to [25].

### 2.4. Preparation and Quality Control of ^68^Ga-NeoBOMB1

^68^Ga-NeoBOMB1 was prepared via a kit-based preparation as described in [27], the NeoBOMB-kit was supplied by GiPharma (Saluggia, Italy). A ^68^Ge/^68^Ga-generator (1850 MBq reference activity, GalliaPharm, Eckert & Ziegler Radiopharma, Berlin, Germany) was used for provision of a ^68^Ga-solution for radiolabeling. Quality control included radiochemical purity testing by ITLC and pH measurements (accepted range 3.2–3.8).

### 2.5. Immunohistochemistry

In accordance with a previous study in GIST cell lines [24], for detection of Gastrin-releasing peptide receptor (GRPR) expression, a polyclonal-rabbit antibody (#PA5-26791, ThermoFisher SCIENTIFIC, Vienna, Austria) was used on tissue samples from four primary tumors and two secondary lesions available prior to study inclusion. Briefly, 1–2 µm sections of formalin-fixed paraffin-embedded (FFPE) GIST samples were deparaffinized before staining on an automated immunostainer (BenchMark ULTRA, Ventana Medical System, Oro Valley, AZ, USA). Antigen retrieval was conducted by incubation with cell conditioning reagent 1 (CC1, Ventana Medical System, USA) for 36 min at 95 °C. Antibody was diluted to 1:100 and was incubated for 32 min. Visualization was done by Ultra View DAB Detection Kit (Ventana Medical System, USA) according to manufacturer’s recommendation. Slides were counterstained with Mayer’s Hematoxylin and mounted using Tissue Tec SCA automated film cover slipper (SAKURA, Tokyo, Japan).

Quantification of GRPR staining was performed by a trained pathologist, scoring the expression intensity on a three-tiered scale: score 1 (low or moderate intensity in <40% or high intensity in <20% of tumor cells); score 2 (low or moderate intensity in 40–80% or high intensity in 20–50% of tumor cells); and score 3 (low or moderate intensity in >80% or high intensity in >50% of tumor cells).

### 2.6. PET Imaging

Administered activity ranged from 127 to 250 MBq (mean 181.6 ± 33.6 MBq) of ^68^Ga-NeoBOMB1. All scans were performed using a Discovery PET/CT 690 VCT scanner (GE Healthcare, Milwaukee, WI, USA). Six patients were scanned with the following imaging protocol: A 5 × 60 s dynamic PET/CT scan of the upper abdomen in the first five minutes after tracer administration, starting at the time of tracer injection (one bed position with an axial field of view of 15.6 cm), followed by three sequential static PET/CT scans of the whole thorax and abdominopelvic region (three bed positions with two min/bed position) at five, 12, and 19 min post injection (p.i.), and three PET/CT scans from the vertex of the skull to the mid-thigh (7 bed positions, 2 min/bed position) at one, two, and three hours p.i. In total, in each patient, five low-dose CT (LDCT) scans were performed for attenuation correction of the PET emission data (one for the dynamic PET/CT, one for the time points from five to 19 min, and one for each of later time points). The low-dose CT scan parameters using BGE smart mA dose modulation were: 100 kVp, 15–150 mA, noise index 60, 0.8 s per tube rotation, slice thickness 3.75 mm and pitch 1.375. Three patients underwent PET scans only at 2 h and 3 h post injection (including two LDCTs), otherwise adhering to the imaging parameters described above.

PET images were reconstructed based on an ordered-subset expectation maximization (OSEM) algorithm with 2 iterations and 24 subsets for static images and 32 subsets for dynamic images. PET image normalization to units of Bq/mL was performed by correcting for sensitivity, attenuation, scatter, dead-time, random coincidences, and for decay to the start of the image acquisition.

### 2.7. CT Imaging

A diagnostic whole-body CT (thorax and abdomen/pelvis 40–70 s after injection and separate thorax in deep inspiration) was acquired after the final whole-body PET scan after body-weight-adjusted intravenous application (1.5 mL/kg body weight [BW]) of Iopromid (Ultravist 370; Bayer AG, Leverkusen, Germany) in case of no recent diagnostic CT of the trunk. Scan parameters were 100–120 kVp, 80–450 mA, noise index 24, 0.8 s per tube rotation, slice thickness 3.75 mm, and pitch 0.984 with BGE smart mA dose modulation. Lesion volume was measured by manually drawn volumetric ROIs. Absolute lesion contrast enhancement was calculated by subtracting native low-dose CT lesion density in Hounsfield units (HU) from post-contrast values, a marker considered to correlate with vitality [26].

### 2.8. PET/CT Image Analysis

PET/CT images were analyzed with a dedicated software (General Electric Advance Workstation SW Version 3.2.2). For visual assessment of PET images, any lesion differing from normal tissue background activity that could not be explained by physiologic tracer uptake or lack thereof (i.e., liver cysts) was rated as suspicious of malignancy. For quantification of tracer activity, maximum standardized uptake values (SUV_max_) in lesions considered malignant on PET or on diagnostic CT was measured, applying volumes of interest (VOIs) that were drawn automatically with a manually adapted isocontour threshold centered on the lesions of interest. In case of PET-negative lesions, manual co-localization was performed in consensus by a specialist in nuclear medicine (C.U.) and in radiology (L.G.). In addition, SUV_max_ of blood pool in the descending abdominal aorta was determined, serving as a reference region. Time activity curves were generated from SUV_max_ values of different time points of image acquisition. Diagnostic CT images were analyzed with Agfa Impax EE (Version R20 XVII SU2; Agfa Healthcare, Mortsel, Belgium) to measure tumor volumes, diameters, and density prior and post contrast agent application.

### 2.9. Statistics

All data were collected and stored in Microsoft Excel (Microsoft Corporation; Redmond, WA, USA). Statistical analysis was carried out in GraphPad Prism 8.4.2 (GraphPad Software Inc.; La Jolla, CA, USA) and SPSS Statistics 24.0 (IBM Corporation; Armonk, NY, USA).

Continuous data are presented as dot plots, including mean and standard deviation (SD), or box plots, including median, 5th, 25th, 75th, and 95th percentiles.

Per-patient tumor detection was classified as uniformly positive, mixed, or negative. Tumor uptake was analyzed on a per-lesion basis, including affected organ, SUV_max_ for all available time points (5, 12, 19 min; 1, 2, and 3 h p.i. for the first 6 participants; 2 and 3 h p.i. for the last 3 participants). Further covariates included patient age, sex, BMI (body mass index), injected activity/kg body weight, disease duration, disease state (progressive, stable, regressive), first-line treatment, mutational status (PDGF-Rα, tyrosine kinase receptor), lesion site, volume, necrotic appearance (defined as rim-like CT contrast enhancement), and overall contrast uptake compared to non-contrast scans (in Hounsfield units).

Overall lesion detection rates of CT and PET-CT (compared against the sum of all lesions detected with either modality), including the agreement rate, were calculated. Furthermore, the detection rate of lesions with necrotic and non-necrotic appearance (in case of any kind of CT contrast uptake, i.e., vital lesions according to Choi et al. [26]), was calculated.

To assess the influence of the aforementioned covariates on ^68^Ga-NeoBOMB1 2h-SUV_max_, a linear regression per-lesion analysis was performed in continuous variables (results presented as slope ± standard error, R^2^, *p*-values) and an ordinary one-way ANOVA with a Holm-Sidak correction for multiple testing in categorical variables (results presented as mean ± SD, 95% CI and *p*-values). Continuous variables were log-transformed to achieve a Gaussian distribution (as assessed by a D’Agostino & Pearson test).

Subgroup analyses were then carried out in participants with confirmed moderate or strong GRPR expression, grouped by lesion location and compared by Fisher’s exact test and χ^2^ test, respectively; results are given as *p*-values.

## 3. Results

### 3.1. Participants

The average age was 64.4 ± 11.2 years; six out of nine participants were female (66.7%). The average time from first diagnosis was 6.9 ± 5.9 years. Five participants had a primary tumor of the small bowel (55.6%); three of the stomach (33.3%); and one of the peritoneum (11.1%). Five out of nine participants (55.6%) presented with progressive disease whereas four patients had stable disease (44.4%). Three participants received first-line treatment (i.e., Imatinib); four participants received second-line treatment (Sunitinib); and one participant received third-line treatment (Nilotinib). Due to an exon 18 D842V of PDGFRa mutation in participant 05, no TKI was used. Further details on demographics and disease state are summarized in Table 1.

### 3.2. Tumor Detection with ^68^Ga-NeoBOMB1 PET/CT

Six of nine participants presented at least one ^68^Ga-NeoBOMB1-PET positive lesion: tracer uptake was shown in all lesions in 3 patients, while only some lesions exhibited uptake in the other 3 patients.

Three of nine patients did not present tracer uptake in tumor lesions visible on CT. For a graphical overview of individual PET/CT imaging results, please refer to Supplemental Figure S1.

In a per-lesion analysis, 37 lesions were identified either by contrast-enhanced CT or ^68^Ga-NeoBOMB1 PET/CT: 64.9% (n = 24) were located in the liver; 8.1% (n = 3) in the stomach; 5.4% (n = 2) in the lung; 5.4% (n = 2) along the peritoneum; 13.5% (n = 5) in the mesentery; and 2.7% (n = 1) in the abdominal wall. Lesions consideredlocal recurrence were 8.1% (n = 3), and the remaining lesions (91.9%) metastases.

^68^Ga-NeoBOMB1 PET/CT identified 17 lesions (45.9%) and contrast-enhanced CT 32 lesions (86.5%) with an overall agreement rate of 32.4% (12 out of 37 lesions). Five lesions (13.5%) were identified only by ^68^Ga-NeoBOMB1 PET, with no correlation on contrast-enhanced CT (Table 2).

On the overall 37 lesions identified, 11 were non-necrotic and 16 presented necrotic aspects at contrast-enhanced CT. ^68^Ga-NeoBOMB1 PET/CT detected 8 out of 11 (72.7%) non-necrotic lesions and only 4 out of 16 (25.0%) necrotic lesions (*p* = 0.022).

### 3.3. ^68^Ga-NeoBOMB1 Tumor Uptake

Overall, all PET-positive lesions (n = 17) showed a median SUV_max_ of 6.6 (range 1.0 to 53.8) over time.

**Figure 1:**
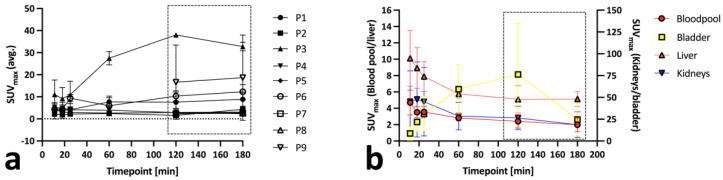
Per-patient ^68^Ga-NeoBOMB1 (**a**) and blood pool, bladder, liver, and kidneys time activity curves (**b**) for all nine participants (P1–9). Optimal imaging window for maximum tumor-to-blood pool contrast is represented by the dashed rectangular box (**a**).

Almost all lesions became visible at 1 h p.i. showing a median SUV_max_ of 7.7 at 1 h p.i. (range 2.4 to 29.6), a median SUV_max_ of 11.8 at 2 h p.i. (range 2.8 to 51.1) and a median SUVmax of 13.2 at 3–4 h p.i (range 2.5 to 53.8). Most positive lesions showed an increase in SUV_max_ over time (Figure 1a,b). Overall, ΔSUV_max_ was highest at 2 h p.i., where the signal-to-blood pool ratio was also highest (dashed box, Figure 1a). An overview over blood pool, bladder, liver, and kidney time-activity curves is given in Figure 1b.

### 3.4. Covariates of Tumor PET Uptake

There was a significant difference in SUV_max_ 2 h p.i. between lesions in female (SUV_max_ 3.14 ± 2.16 [95% CI 2.19 to 4.10]) and male participants (SUV_max_ 18.31 ± 15.23 [95% CI 9.88 to 26.74]; *p* < 0.0001). No significant difference was found for a progressive disease state (SUV_max_ 5.33 ± 4.61 [95% CI 3.04 to 4.61] vs. 13.04 ± 15.78 [95% CI 5.43 to 20.64]; *p* = 0.381), first-line treatment (SUV_max_ 8.93 ± 13.06 [95% CI 2.82 to 15.04] vs. 9.71 ± 11.59 [95% CI 3.75 to 15.67]; *p* = 0.657) or hepatic localization (SUV_max_ 7.69 ± 10.20 [95% CI 3.38 to 11.99] vs. 12.25 ± 15.35 [95% CI 2.97 to 21.53]; *p* = 0.657).

**Figure 2:**
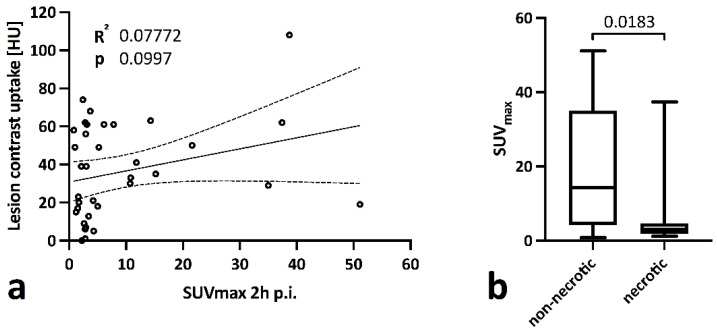
Linear correlation analysis between lesion SUV_max_ (2 h p.i.) and CT contrast-enhancement in all lesions (**a**) and group comparison between lesions with non-necrotic and necrotic CT appearance via a Kruskal-Wallis test (**b**).

When analyzing all lesions, no significant correlation between CT contrast-enhancement and SUV_max_ (0.58 ± 0.34, R^2^ 0.07, *p* = 0.100) was observed, explained by a significantly lower SUV_max_ in necrotic lesions (SUV_max_ 5.20 ± 8.70 [95% CI 0.56 to 9.83] vs. 18.49 ± 16.51 [95% CI 7.40 to 29.58]; *p* = 0.018) Figure 2a,b).

Age (slope 0.07 ± 0.15, R^2^ 0.01, *p* = 0.650), disease duration (slope 0.24 ± 1.00, R^2^ 0.01, *p* = 0.812), injected activity/kgBW (slope −0.01 ± 0.01, R^2^ 0.10, *p* = 0.058), and lesion volume (slope 0.34 ± 0.81, R^2^ 0.01, *p* = 0.677) showed no significant correlation with SUV_max_, while BMI (slope 0.17 ± 0.06, R^2^ 0.18, *p* = 0.007) exhibited a significant positive correlation.

### 3.5. Subgroup Analysis in Confirmed GRPR Expression

**Figure 3:**
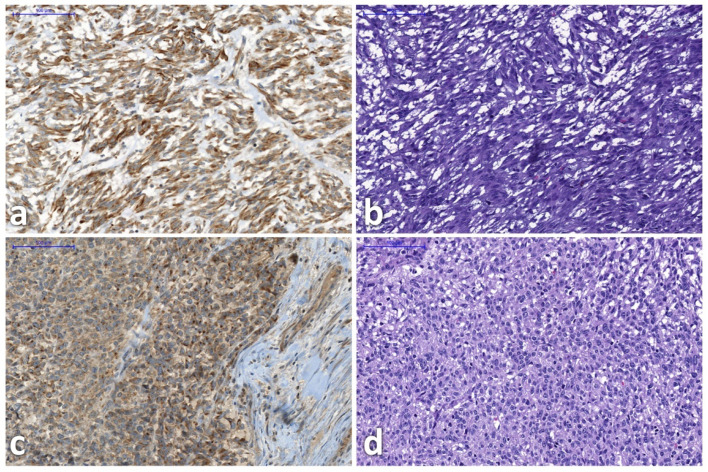
Gastrin-releasing-peptide-receptor immunohistochemistry staining (left side) and correlating hematoxylin-eosin staining (right side) in participants 2 (**a**,**b**) and 3 (**c**,**d**). Images show representative tumor areas. Due to the nature of slide preparation and staining, slides do not fully align.

In six participants GRPR expression tissue sample analysis was available (not acquired within the study): four from primary tumors and two from secondary tumors (exemplary thin-section images after GRP-receptor IHC staining and HE staining provided in Figure 3). This led to an overall of 23 analyzed lesions within PET that could be correlated.

**Figure 4:**
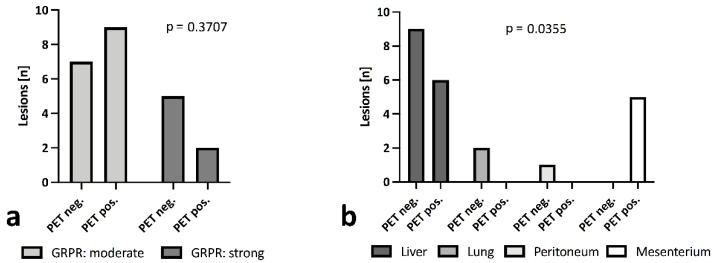
^68^Ga-NeoBOMB1 uptake in lesions grouped by moderate or strong GRPR expression (**a**) and grouped by tumor site (**b**), calculated via Fisher’s exact test and χ^2^ test. All lung lesions appeared calcified, suggesting no vitality.

In all samples, moderate (patients 6, 7, and 9) or strong expression of GRPR (patients 2, 3, and 8) were reported. No significant difference in ^68^Ga-NeoBOMB1 uptake was observed regardless of observed reference lesion GRPR expression (Figure 4a), yet all mesenterial lesions were positive in PET; 40.0% of lesions in the liver; and none in the peritoneum (0%). All lung lesions encountered in a single patient were calcified and showed no tracer uptake (0%) (Figure 4b).

## 4. Discussion

^68^Ga-NeoBOMB1 is a novel PET tracer for the imaging of tumors with gastrin-releasing peptide receptor expression. Its safety, tolerability, and pharmacokinetics have already been demonstrated [25]. In this paper we summarize the final outcome of a Phase I/IIa first-in-human study—conducted within the EU-FP7 project MITIGATE—focusing on tumor-targeting properties of ^68^Ga-NeoBOMB1 in a population of nine participants with advanced, surgically non-curable gastrointestinal stromal tumors. These data complement the first report on safety, tolerability, and pharmacokinetics of ^68^Ga-NeoBOMB1 [25].

The study participants’ demographics are in line with general GIST populations in regard to age and gender distribution, with the diagnosis usually established in their mid-sixties and an equal gender distribution [7]. By study design, and to compensate for an overall low incidence of 4.3 to 22 cases per million [7], inclusion criteria were formulated rather openly. Factors such as disease extent, progression, and interval since first diagnosis showed some variability, also reflecting a new reality of increased survival in GIST due to advances in surgical and pharmacological treatment strategies. Current treatment was also non-uniform with some participants receiving first-line treatment, i.e., imatinib, some second- or third-line treatment. One participant was not treated by any TKI due to an exon 18 D842V of PDGFRa mutation.

**Figure 5:**
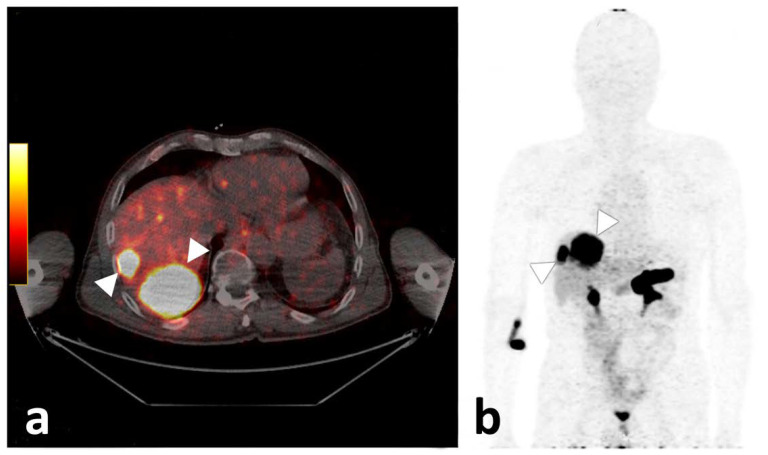
Axial fused PET/CT image of participant 3, demonstrating two liver metastases with high tracer uptake (**a**). On the (**b**), the corresponding maximum intensity projection is given, also demonstrating strong physiologic pancreatic as well as rectal and diffuse small bowel tracer accumulation.

**Figure 6:**
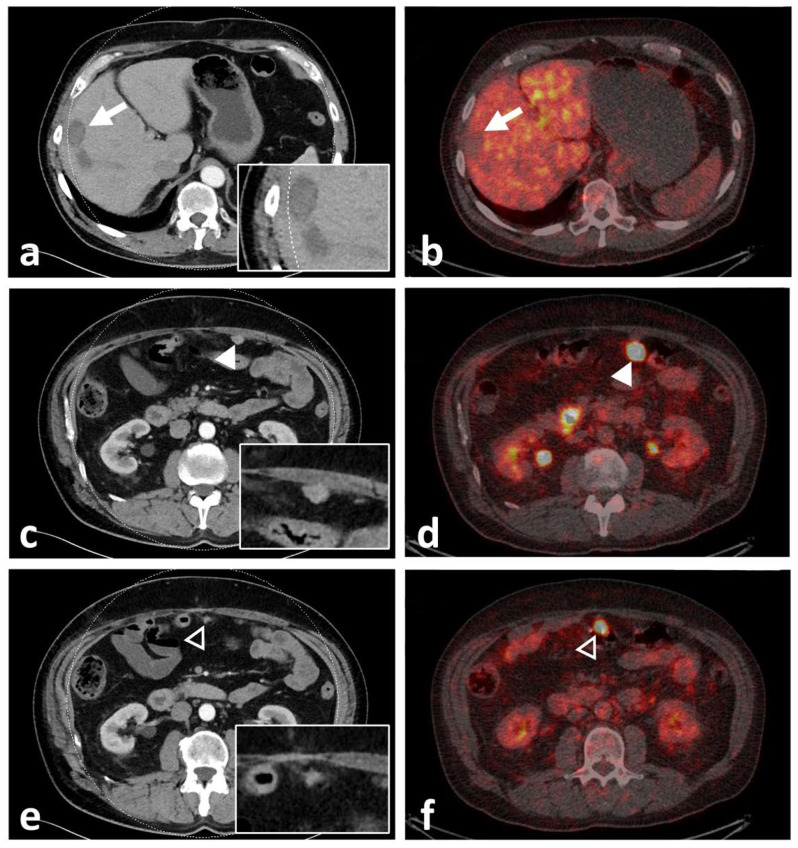
Computed tomography (CT) with inserted magnifications (left) and axial fused PET/CT images (right) of participant 9, demonstrating lack of uptake of ^68^Ga-NeoBOMB1 in a mildly enhancing liver metastasis (white arrow, (**a**,**b**)) and two omental metastases with significant tracer uptake (**c**,**d**) [white arrowhead] and (**e**,**f**) [black arrowhead]). The latter lesion was missed upon initial staging by CT.

Even though physiological ^68^Ga-NeoBOMB1 organ distribution was uniform in all participants with strong uptake mainly in the pancreas and urinary system [25], tumor uptake varied. Tracer accumulation in all lesions was shown in 33.3% (n = 3) of participants: 33.3% (n = 3) showed tracer accumulation only in a subset of lesions; and 33.3% (n = 3) showed no increased uptake in tumor lesions identified on CT (please also refer to the illustrative Figure 5). In three participants, a mixed uptake—i.e., a subset of lesions with no tracer uptake in the presence of other lesions with tracer uptake—were observed (Figure 6). On a per-lesion basis, 17 of 37 lesions (45.9%) were detected by ^68^Ga-NeoBOMB1, whereas 16 lesions (54.1%) were negative on ^68^Ga-NeoBOMB1 PET, explained in part by a significantly lower tracer uptake in lesions with necrotic CT appearance. On the other hand, five lesions (13.5%) could only be identified via ^68^Ga-NeoBOMB1-PET and were not initially evident on CT. Although the current gold standard for staging GIST is still contrast-based CT including Choi-criteria to report on vitality [26], the decision was made early on to include all GIST lesions found with either CT, ^68^Ga-NeoBOMB1-PET, or a combination of both methods to define the overall disease burden most accurately. Overall, we could corroborate study findings by Dimitrakopoulou-Strauss et al. in patients with GIST using ^68^Ga-BZH_3_, that also showed variable uptake in only seven out of 17 patients and eight out of 30 lesions [14].

Patient sex had a significant influence on tracer uptake, although this is most likely linked to the overall low participant number and intraindividual correlations in tumor biology. Nonetheless, potential implications include differing tracer utility based on patient sex. Further studies with larger cohorts should elucidate such correlations. BMI also showed a positive correlation with lesions SUV_max_, while all other assessed parameters—age, disease duration, progressive disease state, first-line treatment, lesion localization, and lesions volume—exhibited no decisive correlation with tracer uptake, in part mirroring previous study results demonstrating stable GRPR expression in GIST cells regardless of disease stage or treatment [15]. In six of our participants, an IHC analysis of existing tissue samples for GRPR expression was available or was performed before study inclusion with all samples showing moderate to high expression patterns. Interestingly, the IHC-confirmed GRPR expression strength did not correlate to lesions SUV_max_; neither did current treatment, disease duration, or progressive disease state. As tissue sampling was not viable within the study, though, IHC was performed on pre-existing tissue samples, which in some cases were several months or years old. Thus, tissue samples may not have been fully representative of the current receptor expression in all lesions. The reason for a heterogeneous receptor expression or a lack thereof in some patients is unclear. Tumor cells may exhibit a reduction in receptor density or mutations regarding the binding site for ^68^Ga-NeoBOMB1 over time, potentially linked to prolonged survival following current treatment regimes. Whether loss of GRPR expression as indicated by low or absent ^68^Ga-NeoBOMB1 uptake is a sign of tumor dedifferentiation and worsening in prognosis remains unclear at this point but may warrant further investigation in larger cohort. Furthermore, ^68^Ga-NeoBOMB1 uptake did not correlate with lesion contrast enhancement (R^2^ 0.04, *p* = 0.24), one of the most widely acknowledged hallmarks of tumor vitality for diagnosis and response evaluation in GIST [26,28]. Only non-hepatic lesion localization showed a weak correlation with uptake intensity. Our results appear to contradict previously reported findings of stable receptor status regardless of size, mitotic index, tumor necrosis, TKI therapy, or chemoembolization [15] as a loss of GRPR receptor density in GIST tissue not linked to a loss of tissue vitality was apparent; its causality is not sufficiently determinable in our small study cohort.

The present pilot study was conducted with a potential radiotherapeutic application in mind. Treatment may be a suitable option in a subset of patients with sufficient ^68^Ga-NeoBOMB1 tumor uptake. As described before, strongest ^68^Ga-NeoBOMB1 uptake occurs in the pancreas at a reported average SUV_max_ of 48.8 ± 20.4 and a dose estimation of 0.27 ± 0.10 mSv/MBq, while the kidneys exhibited comparable SUV_max_ at 47.7 ± 38.1, yet at lower dose estimate of 0.05 ± 0.01 mSv/MBq [25]. As discussed before, pancreatic damage is very unlikely at such doses [29]. Co-administration of stabilizing agents such as peptidase inhibitor phosphoramidon have been suggested to increase tumor-to-organ ratios by increasing peptide stability [30], yet this may not be applicable for ^68^Ga-NeoBOMB1 in face of the already high in vivo plasma stability [25,31].

There are some limitations to this pilot study. Owing to a difficult recruiting process due to low incidence and prevalence as well as severe illness in some potential participants, the cohort size was limited. Even though reflecting prevalent case properties, the study population was heterogeneous in several aspects including disease duration and treatment strategy. IHC was not available in all participants and was conducted in tissue samples sometimes taken several months or years prior to the current study.

## 5. Conclusions

^68^Ga-NeoBOMB1 is a novel GRPR radioligand suited for PET imaging and may deliver information on tumor vitality and burden in GIST patients and may increase sensitivity compared to contrast-enhanced CT. In patients with TKI resistance without other treatment options and uniform ^68^Ga-NeoBOMB1 uptake, a NeoBOMB1-based radioligand therapy may constitute a feasible approach, even if further investigations are needed.

## Figures and Tables

**Table 1:** Patient characteristics and injected activity.

Participant	Age [Years]	Sex	Primary	First Diagnosis	Status at Time of Study	Disease State	Mutational Status	Treatment at Time of Study	Injected Activity [MBq]
01	76	f	Duodenum	2003	Omental and liver metastasis	PD	Exon 11 of cKit gene, TRP 557 ARG	Sunitinib	179
02	72	f	Ileum	2012	Liver metastases	PD	Exon 9 cKit gene	Sunitinib	127
03	72	m	Duodenum	2016	Liver and lung metastases	SD	Exon 9 cKit gene	Sunitinib	214
04	83	f	Stomach	2014	Liver metastases	SD	Exon 11 del557-558 Silent mutation PDGFRa P567P, CCA > CCG	Imatinib	158
05	50	f	Stomach	2016	Local relapse, liver and peritoneal metastases	PD	Exon 18 D842V of PDGFRa gene	None	169
06	55	m	Ileum	2014	Liver metastasis	PD	Exon 11 of cKit gene, secondary exon 17 mutation	Sunitinib	199
07	56	f	Peritoneal	2015	Peritoneal and liver metastases	PD	Exon 11 of cKit gene, PDGF-R wild type	Imatinib	177
08	56	f	Stomach	2001	Liver, lung	SD	cKit-positive, specific mutation not assessed	Nilotinib	161
09	56	m	Small gut	2004	Liver, peritoneal metastases	SD	Exon 9 of cKit gene, PDGF-R wild type	Imatinib	250

SD—stable disease, PD—progressive disease. Further data on safety, tolerability and pharmacokinetics in participants 1–6 were previously reported in [25].

**Table 2:** CT and ^68^Ga-NeoBOMB1 lesion detection rates.

Participant	Overall Lesion Count (CT and/or ^68^Ga-NeoBOMB1 PET/CT)	CT Positive [n/%]	^68^Ga-NeoBOMB1 PET/CT Positive [n/%]	Overlap [n/%]
01	2	2 (100.0)	2 (100.0)	2 (100.0)
02	1	1 (100.0)	0 (0.0)	0 (0.0)
03	2	2 (100.0)	2 (100.0)	2 (100.0)
04	4	4 (100.0)	1 (25.0)	1 (25.0)
05	8	8 (100.0)	3 (37.5)	3 (37.5)
06	4	2 (50.0)	4 (100.0)	2 (50.0)
07	3	3 (100.0)	0 (0.0)	0 (0.0)
08	4	4 (100.0)	0 (0.0)	0 (0.0)
09	9	6 (66.6)	5 (55.5)	2 (22.2)

## Data Availability

Due to privacy and legal reasons, data is not publicly available.

## Supplementary Materials

The following are available online at https://www.mdpi.com/article/10.3390/biomedicines10112899/s1, Figure S1: Mixed ^68^GaNeoBOMB1 uptake.
