# Supplementary material for: Imaging Properties and Tumor Targeting of 68Ga-NeoBOMB1, a Gastrin-Releasing Peptide Receptor Antagonist, in GIST Patients
Source: Biomedicines. 2022 Nov 11;10(11):2899. doi: 10.3390/biomedicines10112899 (PMC9687401; doi:10.3390/biomedicines10112899)
Supplement: Supplementary file 1 [file Supplemental Figure S1.pdf › Supplemental Figure S1.pdf]

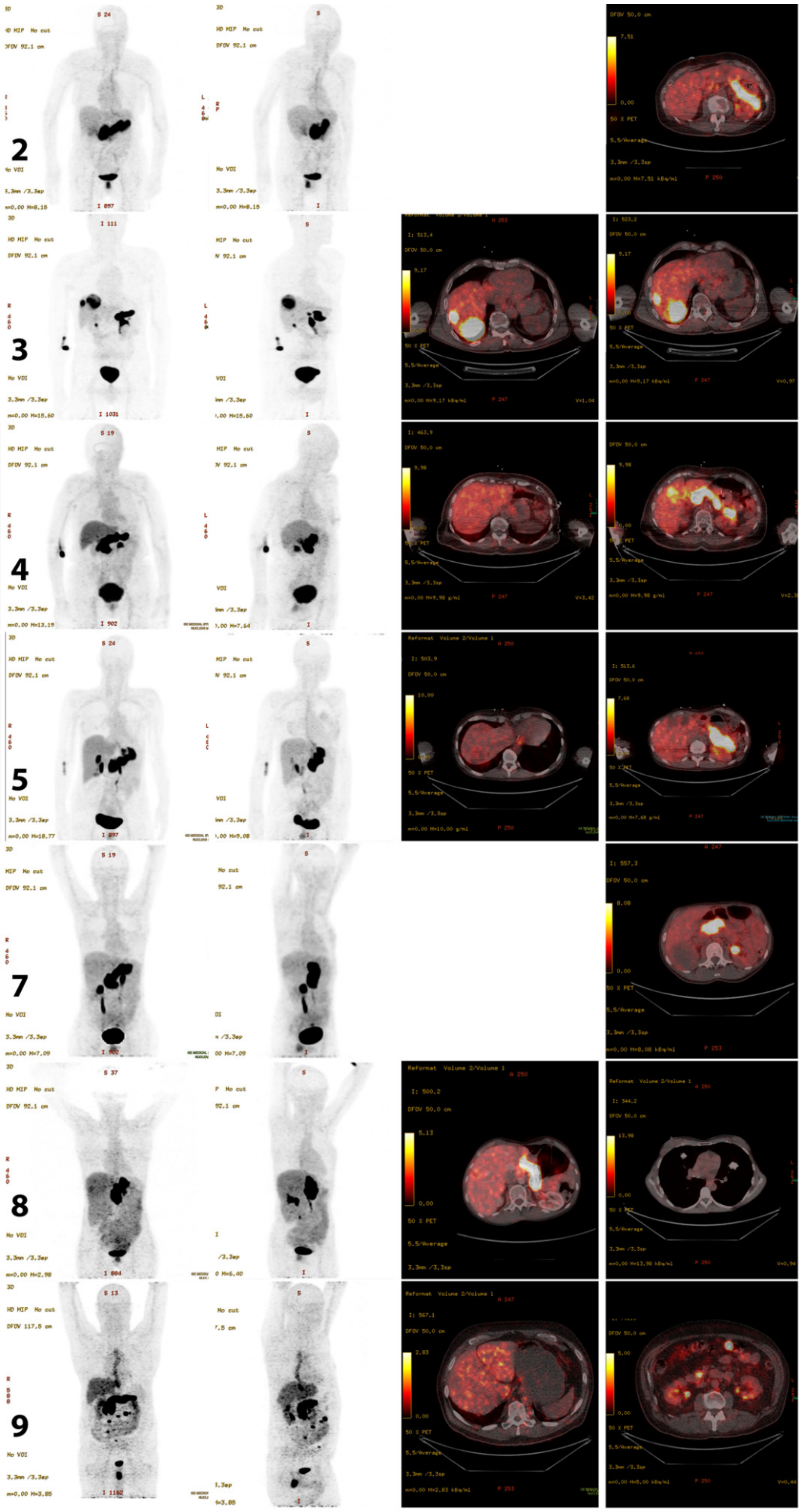

Supplemental Figure S1: Overview of participants 2-5 and 7-9 (row numbers; images of participants 1 and 6 previously published in [25]) including coronal and oblique standard projections angled 45 degrees (first two columns) as well as exemplary axial fused PET/CT images (last two columns) 2h after ^{68}Ga-NeoBOMB1 administration.
